# Coiled coils 9-to-5: rational *de novo* design of α-helical barrels with tunable oligomeric states†

**DOI:** 10.1039/d1sc00460c

**Published:** 2021-04-13

**Authors:** William M. Dawson, Freddie J. O. Martin, Guto G. Rhys, Kathryn L. Shelley, R. Leo Brady, Derek N. Woolfson

**Affiliations:** School of Chemistry, University of Bristol Cantock's Close Bristol BS8 1TS UK w.dawson@bristol.ac.uk d.n.woolfson@bristol.ac.uk; Department of Chemistry, University of Bayreuth, Universitätsstraße 30 95447 Bayreuth Germany; School of Biochemistry, University of Bristol Biomedical Sciences Building, University Walk Bristol BS8 1TD UK; Bristol BioDesign Institute, University of Bristol Life Sciences Building, Tyndall Avenue Bristol BS8 1TQ UK

## Abstract

The rational design of linear peptides that assemble controllably and predictably in water is challenging. Short sequences must encode unique target structures and avoid alternative states. However, the non-covalent forces that stabilize and discriminate between states are weak. Nonetheless, for α-helical coiled-coil assemblies considerable progress has been made in rational *de novo* design. In these, sequence repeats of nominally hydrophobic (**h**) and polar (**p**) residues, **hpphppp**, direct the assembly of amphipathic helices into dimeric to tetrameric bundles. Expanding this pattern to **hpphhph** can produce larger α-helical barrels. Here, we show that pentameric to nonameric barrels are accessed by varying the residue at one of the **h** sites. In peptides with four L/I–K–E–I–A–x–Z repeats, decreasing the size of Z from threonine to serine to alanine to glycine gives progressively larger oligomers. X-ray crystal structures of the resulting α-helical barrels rationalize this: side chains at Z point directly into the helical interfaces, and smaller residues allow closer helix contacts and larger assemblies.

## Abstract 2

Systematic *de novo* design of peptides that form α-helical barrels with functionalisable central channels with a range of internal diameters.

## Graphical abstract

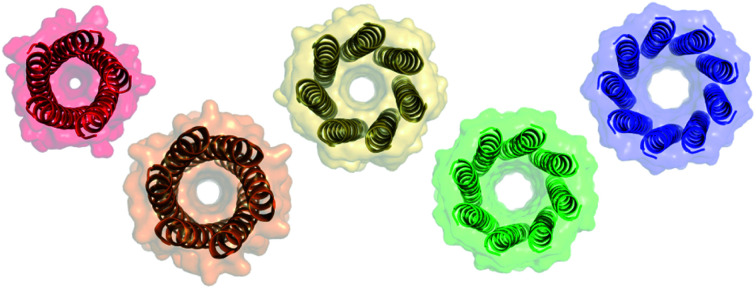

## Introduction

Most commonly, natural coiled-coil (CC) peptides form dimers, trimers and tetramers with consolidated hydrophobic cores.^1,2^ Control over oligomeric state is achieved by different combinations of mainly isoleucine (Ile, I) and leucine (Leu, L) residues in the core.^3,4^ Larger oligomers are rare in nature.^5,6^ Interestingly, some of these larger structures are α-helical barrels (αHBs) with accessible central channels making them appealing scaffolds for functional design, *e.g.* binding, catalysis, delivery, and transport.^7–13^ Variants of a natural dimer and *de novo* tetramer serendipitously form heptameric and hexameric αHBs, respectively.^14,15^ To automate the design of αHBs, we have developed computational-design tools to deliver 5-, 6- or 7-helix αHBs.^16^ These oligomers can be rationalized retrospectively to advance further sequence-to-structure relationships for CC design.

**Fig. 1:**
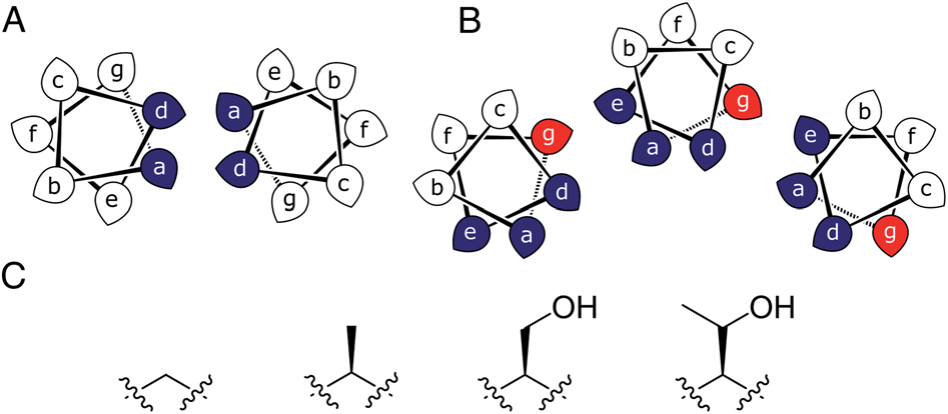
(A and B) Type-N (A) and Type-2 (B) CC interfaces. The **g** position is highlighted in the Type-2 interface. (C) Side-chain structures of glycine, alanine, serine and threonine.

Most αHBs are Type-2 CCs based on **hpphhph** sequence repeats, labelled **abcdefg** (Fig. 1).^17^ Typically, αHBs have L/IxxIAxA repeats; *i.e.*, **a** = Leu or Ile and **d** = Ile. β-Branched residues at **d** are particularly important for maintaining open αHBs.^18^ The hexameric and heptameric αHBs (CC-Hex2 and CC-Hept, systematically named CC-Type2-(S_g_L_a_I_d_)_4_ and CC-Type2-(A_g_L_a_I_d_)_4_) have **a** = Leu, **d** = Ile and **e** = alanine (Ala, A), but differ at **g**, which is Ala in the heptamer and the slightly larger serine (Ser, S) in the hexamer (Table 1). Another variant, CC-Pent (CC-Type2-(I_g_L_a_I_d_E_e_)_4_) has Ile at **g**, although it differs from the other examples having **e** = glutamic acid (Glu, E).^16^ A second series with all-Ile cores (**a** = **d** = Ile) has been characterized.^16,18^ In these, another hexamer, CC-Hex3 (CC-Type2-(S_g_I_a_I_d_)_4_), follows the design rules above (Table 1);^16^ and a peptide with Ala at **g** (CC-Type2-(A_g_I_a_I_d_)_4_) forms an octamer when crystallized in the presence of isopropanol.^18^

**Table 1:** Sequences of *de novo* αHBs and summary of biophysical characterization^a^

Heptad repeat (**abcdefg**)	Systematic name	BUDE oligomer score	DPH *K*_D_ (μM)	XRD αHB oligomeric state(s)	AUC	
					SV	SE
LKEIAxT	CC-Type2-(T_g_L_a_I_d_)_4_	5	6.8 ± 1.3	5	4.8	4.8
IKEIAxT	CC-Type2-(T_g_I_a_I_d_)_4_	5	0.84 ± 0.19	5	5.4	5.2
LKEIAxS^16^	CC-Type2-(S_g_L_a_I_d_)_4_	6	1.6 ± 0.2	6	5.7	6.5
IKEIAxS^16^	CC-Type2-(S_g_I_a_I_d_)_4_	6	3.8 ± 0.8	6	6.2	6.1
LKEIAxA^16^	CC-Type2-(A_g_L_a_I_d_)_4_	7	1.3 ± 0.3	7	6.9	7.0
IKEIAxA^16,18^	CC-Type2-(A_g_I_a_I_d_)_4_	6	2.2 ± 0.3	8	6.1	5.7
LKEIAxG	CC-Type2-(G_g_L_a_I_d_)_4_	9	0.076 ± 0.0065	9 (& collapsed 6)	6.5	6.3
IKEIAxG	CC-Type2-(G_g_I_a_I_d_)_4_	8	0.66 ± 0.12	6 & 7	5.0	5.1

a BUDE: Bristol University Docking Engine. DPH: diphenylhexatriene. XRD: X-ray diffraction. AUC: analytical ultracentrifugation. SV: sedimentation velocity. SE: sedimentation equilibrium.

These previously described αHBs show a trend: increasing the size of side chains at **g** decreases the oligomer state formed. Therefore, we reasoned that a series of minimal changes, solely at the **g** position—*i.e.*, at Z in L/IxxIAxZ repeats—might direct oligomeric state systematically and reliably.

Specifically, we considered the addition of a single heavy atom (C or O) to the side chain through the series glycine (Gly, G), Ala, Ser and threonine (Thr, T) (Fig. 1C). Our aim was to make a series of peptides with minimal changes to give a robust family of αHBs with tuneable oligomeric states for applications in synthetic biology and protein design.^13^ N.B. To maximise further uses of the peptide assemblies, we avoided larger hydrophobic residues at **g**, *e.g.* valine, as our experience is that these can lead to difficulties in purification. We also avoided cysteine because of potential issues with redox chemistry and to reserve it for introducing function in follow-on studies.^11,19,20^

## Results and discussion

To supplement foregoing designs with Ser and Ala at **g**, we designed four peptides with Gly or Thr at this position; *i.e.*, CC-Type2-(G_g_L_a_I_d_)_4_, CC-Type2-(G_g_I_a_I_d_)_4_, CC-Type2-(T_g_L_a_I_d_)_4_ and CC-Type2-(T_g_I_a_I_d_)_4_, Tables 1 and S2.† For simplicity, we refer to these as Gly@**g** and Thr@**g** peptides. Our hypothesis was that these should direct larger and smaller oligomers, respectively.

We built and optimized parametric models for Gly@**g** and Thr@**g** in ISAMBARD.^21^ The sequences were modelled as parallel αHBs of oligomer state 5 to 10, and scored using BUDE^22,23^ (Table S3†). For the historical designs, the most-favoured states were indeed those observed experimentally,^16^ the exception being CC-Type2-(A_g_I_a_I_d_)_4_, which predicted as a hexamer as observed in solution, but crystallized as an octamer.^18^ Encouragingly, the new Thr@**g** sequences consistently scored best as pentamers: for the **a** = **d** = Ile variant the pentameric assembly was favoured outright; while the **a** = Leu, **d** = Ile variant scored equally well as pentamer or hexamer. Conversely, both Gly variants scored more favourably as larger oligomeric states: the **a** = **d** = Ile variant as an octamer; and **a** = Leu, **d** = Ile as a nonamer. Although, there was less discrimination between models for the Gly@**g** sequences than for Thr@**g**, Fig. S1.† Thus, modelling supports the hypothesis that oligomeric states of Type-2 CCs can be tuned by side chains at **g**.

**Fig. 2:**
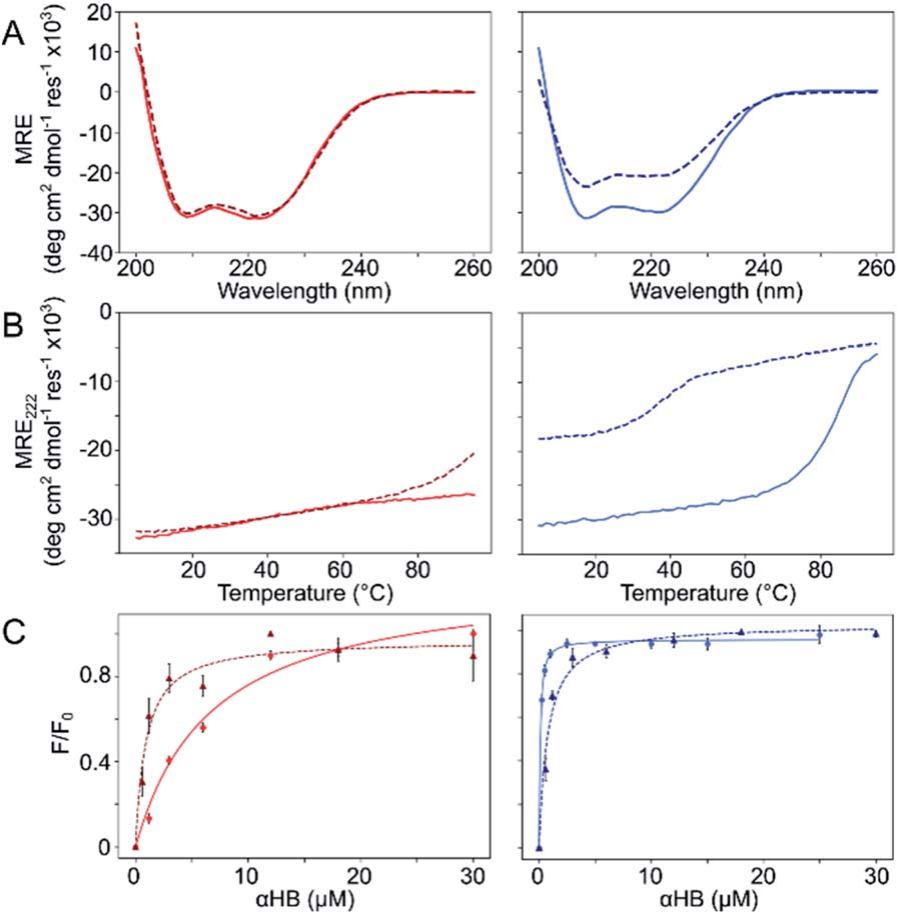
Solution-phase biophysical characterization of Gly@**g** (right) and Thr@**g** (left) peptides. (A) CD spectra at 20 °C. (B) Thermal denaturation following the CD signal at 222 nm (MRE_222_). (C) Saturation binding curves with DPH. Key: CC-Type2-(T_g_L_a_I_d_)_4_ (red), CC-Type2-(T_g_I_a_I_d_)_4_ (dark red, dashed), CC-Type2-(G_g_L_a_I_d_)_4_ (blue) and CC-Type2-(G_g_I_a_I_d_)_4_ (navy, dashed). Conditions: (A and B) 10 μM peptide. (C) 0–300 μM peptide, 1 μM DPH, 5% v/v DMSO. All experiments were performed in phosphate buffered saline at pH 7.4 (PBS; 8.2 mM Na_2_HPO_4_, 1.8 mM KH_2_PO_4_, 137 mM NaCl, 2.4 mM KCl).

The Gly@**g** and Thr@**g** peptides were synthesized, purified by HPLC, and confirmed by mass spectrometry (Fig. S2–S8†). Circular dichroism (CD) spectroscopy indicated that all four peptides were α helical at low μM concentrations, Fig. 2A. CD spectra recorded at increasing temperatures showed that both Thr@**g** variants were hyperthermostable, Fig. 2B. Whereas, the Gly@**g** variant with **a** = Leu, **d** = Ile had the beginnings of a thermal unfolding curve consistent with the anticipated destabilizing effect of Gly on α-helical structures.^24–26^ The Gly@**g** variant with **a** = **d** = Ile unfolded at 38 °C and did not fully refold on cooling. Indeed, peptide sequences with **a** = **d** = Ile are more unstable than their **a** = Leu, **d** = Ile counterparts. Whilst β-branched residues like Ile clearly impart oligomer-state specificity in CC systems,^3,4,18^ we posit that this may be at the expense of some loss in overall thermal stability.

Next, dye-binding assays were used to assess the presence of accessible channels, Fig. 2C and S9† and Table 1. Binding of diphenylhexatriene (DPH) is a reliable indicator that CC peptides form αHBs in solution with a 1 : 1 correlation with X-ray crystal structures.^16,18^ All four new peptides bound DPH consistent with the formation of αHBs.

**Fig. 3:**
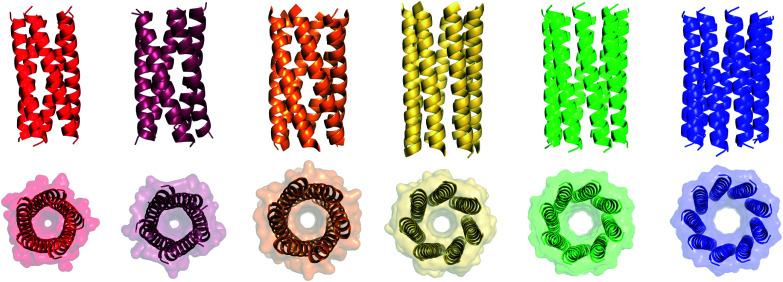
X-ray crystal structures for pentameric through to nonameric αHBs. (Left to right) CC-Type2-(T_g_L_a_I_d_)_4_ (red, PDB 7BAS), CC-Type2-(T_g_I_a_I_d_)_4_ (dark red, PDB 7BAU), CC-Type2-(S_g_L_a_I_d_)_4_ (orange, PDB 4PN9), CC-Type2-(A_g_L_a_I_d_)_4_ (yellow, PDB 4PNA), CC-Type2-(A_g_I_a_I_d_)_4_ (green, PDB 6G67) and CC-Type2-(G_g_L_a_I_d_)_4_ (blue, PDB 7BIM).‡‡X-ray crystal structures for CC-Type2-(T_g_L_a_I_d_)_4_-W19BrPhe_,_ CC-Type2-(T_g_I_a_I_d_)_4_-W19BrPhe, CC-Type2-(G_g_L_a_I_d_)_4,_ CC-Type2-(G_g_L_a_I_d_)_4_-W19BrPhe and CC-Type2-(G_g_I_a_I_d_)_4_ are available from the Protein Data Bank. Accession codes: 7A1T, 7BAS, 7BAT, 7BAU, 7BAV, 7BAW & 7BIM.

We determined X-ray protein crystal structures for all Gly@**g** and Thr@**g** variants. As predicted, the Thr@**g** peptides formed parallel pentamers (Fig. 3 and Tables S4 and S5†). Comparison with the foregoing computationally designed pentamer, CC-Type2-(I_g_L_a_I_d_E_e_)_4_,^16^ revealed similar CC parameters for the structures. Significantly, Thr@**g** with **a** = **d** = Ile has a wider channel of ≈9 Å than previous designs (≈7 Å), which increases the scope to functionalize this variant.

**Fig. 4:**
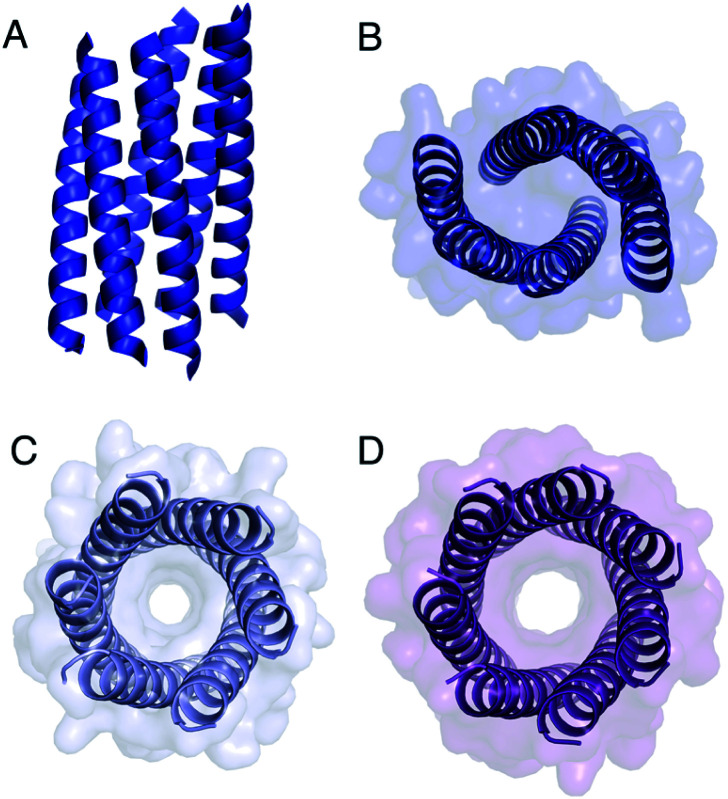
Structures of Gly@**g** variants. (A and B) Orthogonal views of the collapsed hexameric-form of CC-Type2-(G_g_L_a_I_d_)_4_ (PDB 7A1T). (C and D) The hexameric (C) and heptameric (D) form of CC-Type2-(G_g_I_a_I_d_)_4_ (PDB 7BAT & 7BAW).‡

The Gly@**g** peptide with **a** = Leu, **d** = Ile crystallized in two forms. Gratifyingly, one was an all-parallel nonamer, which is a new αHB with an exceptionally large channel of diameter ≈9.5–11.5 Å, Fig. 3 (Table S4†). Attempts to model solvent into density observed in the channel were inconsistent. Therefore, representative solvent molecules were included where they matched the density; though these did not make any stabilizing contacts with protein. Although there are natural nonameric protein assemblies,^27,28^ this is the first stand-alone, water-soluble α-helical CC of this size. The second crystal form revealed a collapsed C2-symmetric 6-helix bundle (Fig. 4A and B, Table S4†). This was surprising, as the β-branched Ile residues at **d** would be expected to prevent collapse.^18^ We posit that the small size of Gly relaxes this design rule allowing access to other nearby regions of the CC free-energy landscape.^29^

Gly@**g** with **a** = **d** = Ile also crystallized in two forms (Fig. 4C and D, Table S5†), However, both solved as αHBs; namely, a hexamer and a heptamer. Thus, β-branched Ile at both **a** and **d** maintains the open assembly^18^ even with Gly residues at **g**. Although a larger oligomer was predicted *in silico*, the calculated internal energies for Gly@**g** were similar for the different oligomers (Fig. S1†).

Because of the apparent structural duality with Gly@**g**, we examined the oligomeric states of all peptides in solution by analytical ultracentrifugation (AUC; Table 1, Fig. S10–S13†). Both sedimentation velocity (SV) and sedimentation equilibrium (SE) measurements for the Thr@**g** variants returned pentameric molecular weights consistent with the X-ray crystal structures. Indeed, for the pentamers through heptamers for these and previous designs, the correlation between the solution-phase and crystal-state oligomers was good, Table 1.

However, where larger oligomers (octamer^18^ and nonamer) were observed in crystals, smaller oligomers were consistently observed in solution, Table 1. This suggests that the solution states are the dominant species, and that the higher oligomers observed by X-ray crystallography are meta-stable. This is consistent with smaller oligomers being entropically favoured. In addition, the crystallization conditions for the octamer and nonamer contained isopropanol. This increases the hydrophobicity of the bulk solvent, which potentially supports larger, hydrophobic pores that would otherwise be energetically unfavourable.^29^ Nevertheless, these are legitimate states to consider as they are clearly accessible on the CC free-energy landscape.

Summarizing these data, Type-2 CC peptides with sequence repeats LppIApZ and Z = Thr, Ser, or Ala, form pentameric, hexameric, and heptameric αHBs, respectively. Adding Gly to the series accesses a nonameric open barrel, but only in the crystal state. Similarly, an IppIApA peptide forms an octamer in the crystal state.^18^ These X-ray crystal structures enabled us to examine the structural transitions in detail.

**Fig. 5:**
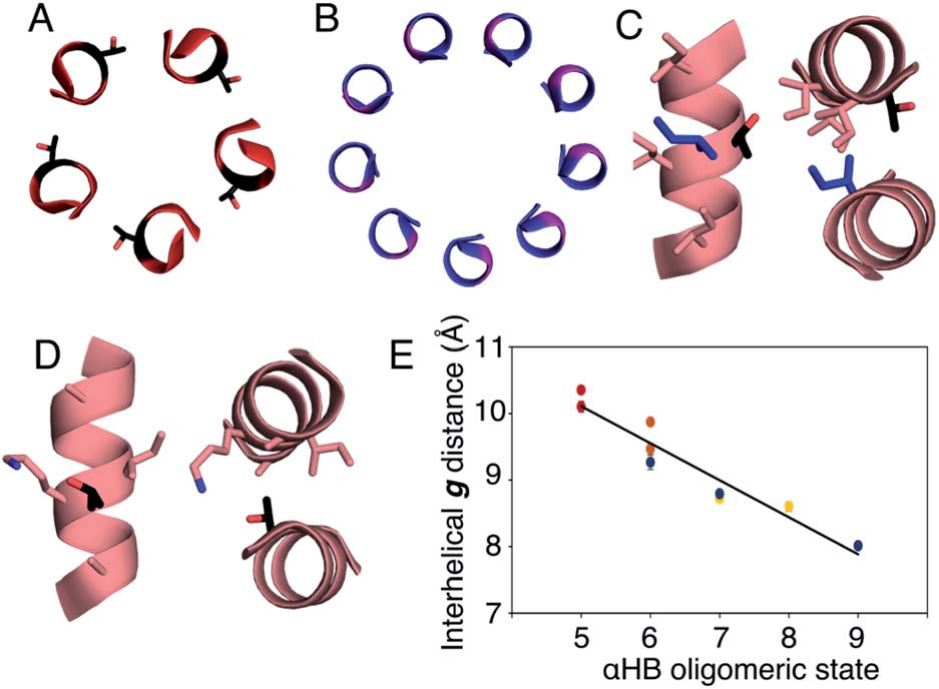
Analysis of pentameric to nonameric αHBs. (A and B) Cross-sections through CC-Type2-(T_g_I_a_I_d_)_4_ (red) and CC-Type2-(G_g_L_a_I_d_)_4_ (blue) showing the geometry of residues at **g** (black and pink, respectively). (C and D) Knobs-into-holes interaction showing how these residues (black) contribute to a **d′-1-g′-1-a′-d′** hole when Ile is the knob residue (blue) (C); and when the Thr at the **g** position is a knob residue (D). (E) Interhelical distances in the αHBs with Thr (red), Ser (orange), Ala (yellow) and Gly (blue) at **g**. Errors are the standard deviation of measurements from the central heptads of each structure.

In all of the structures, the **a** and **d** residues contribute both to the lumens and to interactions between neighbouring helices. SOCKET^30,31^ analysis revealed that residues at **a** form knobs that fit into holes made by **d′-1-g′-1-a′-d′** of a neighbouring helix.^32^ Thus, these knobs are complemented by interactions formed by the residues at **g′-1** varied herein. The Cα-to-Cβ bond vectors of side chains at **g** point directly towards the adjacent helix, Fig. 5A–D. In classical CCs, this is called perpendicular packing, and it restricts how close the helices can approach.^3,32,33^ Thus, mutations at **g** might be expected to influence the quaternary structure. The changes made in the Gly→Ala→Ser→Thr series progressively add a single heavy atom to that side chain: Gly (0 heavy atoms)→Ala (1)→Ser (2)→Thr (3), Fig. 1C. It is gratifying, but still surprising, that this leads to unitary changes in oligomer state, at least for the Ala, Ser, and Thr variants. This is manifest in adjacent helix–helix distances through the series, Fig. 5E. The average distance increases from 8.0 ± 0.1 Å in the Gly@**g** nonamer to 10.4 ± 0.1 Å in the Thr@**g** pentamer. Thus, through the series, neighbouring helices are pushed apart effectively expelling helices from the assembly.

The judicious placement of Gly may prove useful in designing αHBs to unlock previously unseen architectures. However, using Gly presents challenges that must be met to allow its full exploitation. The first challenge is incorporating multistate design into αHBs, *i.e.* considering multiple conformations and/or assemblies that may become accessible in the CC free-energy landscape.^29^ This approach is being applied to other systems.^34–36^ It is tractable to model many possible Type-2 αHBs to direct computational design.^16^ However, this becomes difficult with increasing off-target states, *e.g.* collapsed and anti-parallel structures. The second challenge is to stabilize the larger, but clearly accessible, oligomer states in solution. One possibility would be to introduce networks of polar residues to reduce the penalty of all-hydrophobic channels.^37^ The hexameric and heptameric assemblies of CC-Type2-(G_g_I_a_I_d_)_4_ imply that, whilst Gly@**g** is necessary to access larger states, it is not the only factor that dictates oligomer state of the helical assembly. *De novo* αHBs have proved useful in functional protein design.^11–13^ Reliably accessing scaffolds with significantly larger pores systematically and with minimal changes in primary sequence, would expand the scope for this and future applications.

## Conclusions

We have combined rational design, computational modelling, and structural biology for a series of α-helical barrels (αHBs) with mutations from Gly→Ala→Ser→Thr at all **g** sites of a coiled-coil (CC) sequence repeat. Minimal and stepwise changes in size of the residue at these sites, combined with Leu/Ile at **a** and Ile at **d**, control the oligomeric state of the assembly. This expands the range of αHBs that can be designed systematically from pentamer (with Thr at **g**) to a nonamer (with Gly at **g**). Inspection of X-ray crystal structures rationalizes the role of side-chain bulk at **g** in dictating inter-helical packing distance, angles, and, thus, oligomeric state. CC-Type2-(G_g_L_a_I_d_)_4_ is the first example of a stand-alone nonameric CC. However, it appears that high oligomeric states (such as 8 and 9) are on the edge of what is possible for such ‘Type-2’ CC sequences as they are not favoured in solution.^16,18,38–40^ Nonetheless, the X-ray crystal structures show that they are accessible. The rarity of such assemblies in nature^6,17,27,28^ and their potential as scaffolds for functional *de novo* design makes these large αHBs tantalizing targets for design. For example, *de novo* designed and engineered αHBs have already proven useful in constructing peptide nanotubes,^8,9,12^ peptide-based switches,^10,29^ a rudimentary catalyst,^11^ membrane-spanning ion-channels^41^ and small-molecule receptors.^13^

## Author contributions

WMD and DNW designed the study. WMD performed the biophysical characterisation of all peptides. WMD and FJOM crystallised the peptides. FJOM and GGR collected X-ray crystal data. FJOM, GGR, KLS and RLB determined the peptide crystal structures of the peptides. WMD and DNW wrote the manuscript. All authors read and contributed to the preparation of the manuscript.

## Conflicts of interest

There are no conflicts to declare.

## Supplementary Material

SC-012-D1SC00460C-s001

SC-012-D1SC00460C-s002

SC-012-D1SC00460C-s003

SC-012-D1SC00460C-s004

SC-012-D1SC00460C-s005

SC-012-D1SC00460C-s006

SC-012-D1SC00460C-s007

SC-012-D1SC00460C-s008

SC-012-D1SC00460C-s009

SC-012-D1SC00460C-s010

SC-012-D1SC00460C-s011

SC-012-D1SC00460C-s012

SC-012-D1SC00460C-s013

SC-012-D1SC00460C-s014

SC-012-D1SC00460C-s015

SC-012-D1SC00460C-s016

SC-012-D1SC00460C-s017

SC-012-D1SC00460C-s018

SC-012-D1SC00460C-s019

SC-012-D1SC00460C-s020

SC-012-D1SC00460C-s021

SC-012-D1SC00460C-s022
